# Reframing the banning of flavored tobacco in unprecedented times- an example from California’s Senate bill 793

**DOI:** 10.1016/j.pmedr.2022.101783

**Published:** 2022-04-04

**Authors:** Jasmine Tang Ker, Natalie Delgadillo, Dania Amiri, David S. Timberlake

**Affiliations:** Program in Public Health, College of Health Sciences, University of California, Irvine Anteater Instruction & Research Building, Irvine, CA 92697, USA

**Keywords:** Tobacco flavor bans, Policy, Tobacco industry, COVID-19, Black lives matter

## Abstract

Several cities, but only two U.S states, have passed a law banning the sales of flavored tobacco products. It has been suggested that framing tobacco control policy solely in terms of the youth could send the erroneous message that tobacco use is an acceptable behavior for adults. This study was intended to compare the framing of policy between California’s Senate Bill (SB) 38 and 793. Seven audio files of hearings on SB-38 (N = 2) and SB-793 (N = 5), held between March 2019 and August 2020, were transcribed and coded for youth issues and the unprecedented events of 2020 that shaped society’s views of health and racial/social justice. The Framework Method was used for organizing and analyzing content of the legislative hearings. Many of the same arguments pertaining to youth were presented in hearings on the two bills. The one notable difference was legislators’ sense of obligation to younger constituents, which was expressed in hearings on SB-793, but not SB-38. The hearings on SB-793 also differed with respect to greater discussion about the relevance of a tobacco flavor ban to society as a whole. These discussions revolved around the COVID-19 pandemic and potential impact of a ban on communities of color. Discussions on SB-793 about the larger societal impact of flavored tobacco may be a more effective strategy than focusing exclusively on the youth. Thus, legislators from other U.S. states who are contemplating a statewide ban should consider reframing the issue according to California’s SB-793.

## 1 Introduction

Adolescents’ use of flavored tobacco is an ongoing public health problem that can be attributed in part to perceptions of lower health risk, greater taste and greater appeal (Huang et al., 2017). But, the concern about flavored tobacco also extends to adult tobacco consumers, notably African American menthol smokers (∼90%, 26 to 49-year-olds) (Giovino et al., 2015). The popularity of flavored tobacco among various demographic groups, and the need to address it, has caught the attention of public health officials and legislators who are proposing product restrictions.

On August 28, 2020, Senate Bill (SB) 793, unlike its predecessor SB-38, was passed by the California State Legislature and signed into law (Aguilera, 2020). With the exception of premium cigars, hookah and loose leaf tobacco, SB-793 prohibits the sales of flavored tobacco products and the flavored liquids (e-liquids) used for vaping. A sufficient number of signatures were subsequently garnered to place a veto referendum on the ballot in November 2022 for California voters to determine whether SB-793 would be upheld or overturned. For many outside observers, the passage of the progressive bill may not have come as a surprise. Starting with Proposition 99, which funded the California Tobacco Control Program (CTCP) through a tobacco tax in 1988 (Roeseler and Burns, 2010), the Golden State has been on the forefront of many tobacco control initiatives. It was the first state to enact a comprehensive smoke-free workplace law in 1995, which was preceded by the passage of several ordinances in local jurisdictions. Similar to the workplace ordinances, over 80 California jurisdictions had enacted a partial or comprehensive ban on the sales of flavored tobacco prior to the passage of SB-793 (Initiative, 2019). The ban in San Francisco was exceptional because R.J. Reynolds was successful in forcing a referendum, which, to its dismay, was upheld with the support of most voters (68%) (Yang and Glantz, 2018).

The primary question of the present study is whether the proponents of SB-793, and its predecessor SB-38, framed the flavored tobacco ban as an issue concerning only youth or the general population. An argument for the former is supported by the meteoric rise in adolescents’ use of vaping devices (Miech et al., 2019) which coincided with the introduction of SB-38 in the California State Legislature (December 2018). Yet, it has been suggested that a primary focus on youth in tobacco control policy could convey the message that tobacco use is an acceptable behavior for adults, rather than a behavior that is harmful to all (Menashe and Siegel, 1998). Such a message has been reinforced by the tobacco industry’s support for youth access laws. The current study hypothesizes that the unprecedented events of 2020 (e.g., COVID-19, Black Lives Matter) have shifted the debate over the banning of flavored tobacco sales, in discussions of SB-793 versus SB-38, to an issue that affects most age demographics. The need for such a shift is exemplified by smokers’ greater susceptibility to the adverse outcomes associated with a COVID-19 infection (Vardavas and Nikitara, 2020). Another example is the impact of menthol cigarettes, which are heavily marketed in the African American community (Mills et al., 2021), on young and old smokers alike. In responding to such arguments, the tobacco industry appears to be framing the flavor ban as a discriminatory policy, evidenced by slogans such as “No on SB-793, It’s unfair to communities of color” (Aguilera, 2020). Emphasizing the larger societal impact of flavored tobacco, rather than framing it solely as a youth issue, could have important implications for other U.S. states that are contemplating California’s bold initiative.

## 2 Materials and methods

### 2.1 Legislative hearings

Seven audio files of hearings on SB-38 and SB-793 were obtained from websites of the California State Senate (https://www.senate.ca.gov) and the California State Assembly (https://www.assembly.ca.gov). The hearings on the bills occurred in the Health Committee, Appropriations Committee and the Senate Floor between March 27, 2019 and August 4, 2020. Hearings on SB-38 and SB-793 occurred prior to and during the COVID-19 pandemic, respectively. The audio files were transcribed into approximately 120 pages of text and subsequently coded. Public comments on SB-38 and SB-793 were provided by individuals representing a host of organizations, including the African American Tobacco Control Leadership Council, various health associations (e.g., AHA, ALA, CMA), International Youth Tobacco Control, Vapor Technology Association, JUUL Labs, and other groups. Several private citizens, former smokers, business owners, high school students, academicians, and others provided testimony.

### 2.2 Transcript coding and analysis

Based on our hypothesis, the two broad categories of codes were youth issues and the events of 2020 that shaped society’s view of health and racial/social justice. The codes for the youth issues were the influence of peers; a sense of obligation for future generations; health consequences of using the flavored tobacco products (e.g., vaping-related lung injury); the gateway from e-cigarette/vaping devices to conventional cigarettes; and the marketing and characteristics of flavored products that appeal to youth. The codes pertaining to events of 2020 were the effect of tobacco use on the risk of developing COVID-19 complications and concerns of the African American community. The latter included the discriminatory act of banning menthol cigarettes; criminalizing the sales or possession of flavored tobacco; exposure to tobacco marketing; use of tobacco products; and tobacco-related health inequities, including pre-existing conditions for COVID-19 complications. Other codes not directly related to the study’s hypothesis, albeit important, were harm reduction, personal rights, business/revenue loss, and the trend in passing flavored tobacco ordinances in California jurisdictions.

The Framework Method was used for organizing and analyzing the content of the legislative hearings, which entailed transcribing the audio files, identifying codes, developing and applying an analytical framework, and interpreting the data (Gale et al., 2013). Codes corresponding to the two bills were not compared quantitatively because of non-independent observations and the difference in number of hearings on SB-38 (N = 2) and SB-793 (N = 5). Instead, the former and latter were compared qualitatively to assess whether the framing of supportive arguments shifted from a youth issue to an issue that affected most age groups. The software program Atlas.ti v8.2 was used to organize and manage the data (https://atlasti.com).

## 3 Results

Concerns about the youth, which included the appeal of flavors, the gateway to cigarettes, and youth marketing tactics, were a major theme in discussions of the two bills. Many of the same arguments and statistics pertaining to youth were presented in hearings on SB-38 and SB-793. California State Senator Jerry Hill, author of SB-793 and SB-38, said during one hearing “*Flavors play an outsized and dangerous role in youth tobacco initiation with 80% of youth [who have started] with a flavored product*”. But, unlike the hearings on SB-38, legislators supportive of SB-793 conveyed a sense of obligation to younger constituents. Senator Leyva expressed that the obligation was not being fulfilled by adults, who unlike students, have done nothing about the problem of vaping in high schools. Concerns about the proliferation of flavored e-liquids in schools were highlighted by the students who expressed support during the public hearings for a statewide flavor ban. Discussions did not address the vaping-related lung illnesses that were publicized in the summer of 2019 because the last available hearing on SB-38 occurred in the spring. A secondary argument for supporting SB-38, which was raised only once, was passage of local flavor bans across California jurisdictions; surprisingly, the issue was not addressed in any of the hearings on SB-793.

The hearings on SB-793 took place during onset of the COVID-19 pandemic and worldwide protests for racial justice. These events were affecting society as a whole and were taken into consideration by both proponents and opponents of the bill. For example, the concern about smokers’ susceptibility to COVID-19 complications was frequently raised by proponents, one of whom said “*As we fight coronavirus, it has never been more important to keep our lungs healthy. There is clear evidence that smoking and vaping harm the lungs*”. But, opponents of the bill stated that COVID-19 posed challenges such as the inability to fund enforcement of the flavor ban due to the economic downturn. The debate over banning flavored tobacco also centered largely on concerns of the African American community (Table 1). Proponents of SB-793 frequently argued that African Americans were targeted by the tobacco industry, vulnerable to COVID-19 complications (partly due to smoking), and misinformed about the criminalization of young Black men. Supporters of both SB-38 and SB-793 voiced concerns about the ubiquity of tobacco marketing and availability of discounted menthol cigarettes in communities of color. Opponents expressed that communities of color were being singled out because of their preference for menthol cigarettes and flavored cigars. Others testified about the potential criminalization of individuals who possess or sell flavored tobacco products. Additional opposition came from business owners, legislators concerned about lost tax revenue, and cigarette smokers who wanted a less harmful alternative and right to consume flavored tobacco.

**Table 1:** Number of arguments supporting and opposing legislation banning flavored tobacco from perspective of the African American community.

Argument	Senate Bill 793		Senate Bill 38		Example Quotes from SB-793
*Health* *Inequities*	Support^1^	Oppose^1^	Support	Oppose	Supportive argument *“…a disproportionate number of African Americans, who have been impacted by a virus that attacks the lungs, have pre-existing lung vulnerability as a result of long-term smoking.”*
	5	0	0	0	
*Tobacco* *Marketing*	Support	Oppose	Support	Oppose	Supportive argument *“There are more advertisements, more lucrative promotion, and menthol cigarettes are cheaper in the Black community compared to other communities.”*
	5	0	3	0	
*Use of Tobacco Products*	Support	Oppose	Support	Oppose	Supportive argument *“Menthol cigarettes and flavored little cigars have been and are the main factors of death and disease in the Black, Brown, and other poor communities of color.”* Opposing argument *“This bill will have a negative impact on our communities of color. What we see here is this bill disrespects our community of colors and their preference [for menthol], while it exempts hookah products on behalf of Middle Eastern cultures.”*
	2	2	0	0	
*Criminal-ization*	Support	Oppose	Support	Oppose	Supportive argument *“Some Black groups spurred on by the tobacco industry have been spreading false info. stating that prohibiting menthol will lead to criminalization, particularly of young Black men. Nothing could be further from the truth. SB-793 would prohibit the sale of flavored products, not prohibit possession.”* Opposing argument *“If you put a ban on menthol, it will criminalize this product. It will cause unintended consequences. What do you mean? Have you ever heard of Eric Garner in New York?”*
	1	3	0	0	

1 Corresponds to the number of separate arguments within the same category that were presented by legislators, public health officials, members of the general public, and others.

## 4 Discussion

The findings of this study indicate that youth issues, particularly the appeal of flavors, were a consistent theme used in support of a tobacco flavor ban. The testimonials for SB-38 given by high school students, representing groups such as the International Youth Tobacco Control, contributed to the framing of the policy as protection of the youth. Studies have reported that California youth and young adults have been supportive of strong tobacco control measures (Sonnenberg et al., 2020), including a tobacco flavor ban in jurisdictions that do (48.5%) and do not (51.1%) have such a policy (Feld et al., 2021). While many of the same arguments pertaining to youth were presented in hearings on SB-38 and SB-793, only in the latter did legislators convey a sense of obligation for the younger constituents. This sense of obligation may reflect the concerns of parents (Czaplicki et al., 2020), some of whom are California legislators. (Czaplicki et al., 2020) concluded that parents, 75% of whom support a ban on flavored e-cigarettes, are an understudied but influential group of stakeholders who could advance a progressive agenda for tobacco control policy.

The arguments in support of SB-793 addressed to a greater degree issues relevant to multiple demographic groups, including youth, adult tobacco users, and African Americans.

Hence, the hearings followed the recommendation of framing a tobacco control policy as an issue affecting more than one group (i.e. youth) (Menashe and Siegel, 1998). Consistent with the logic of Menashe and Siegel (1998), a frame that emphasizes the health threat of flavored tobacco to smokers and young non-smokers is more likely to be effective than a frame, for example, that focuses exclusively on marketing tactics of tobacco companies. Our finding that proponents of SB-793 voiced concerns about smokers’ vulnerability to COVID-19 complications exemplifies the importance of highlighting the larger societal impact of flavored tobacco. Such an approach could potentially lead to passage of flavored tobacco bans in other U.S. states, complementing the U.S. Food and Drug Administration’s federal ban on menthol and characterizing flavors in cigars and cigarillos (Denlinger-Apte et al., 2022). Some editorials have taken a step further in viewing the COVID-19 pandemic as a unique opportunity for disbanding the industry (Ioannidis and Jha, 2021). Other arguments for SB-793 addressed the marketing and disproportionate use of certain flavored tobacco products in communities of color, not just the youth in these communities. The counter arguments to SB-793, as they apply to communities of color, bear resemblance to the industry’s framing of tobacco control policy as a violation of freedom, fairness, and civil liberties (Menashe and Siegel, 1998, Cheyne et al., 2014). Similar to our findings, (Cheyne et al., 2014) reported that racial discrimination against African Americans was frequently argued on both sides of the debate over the FDA’s regulation of menthol cigarettes.

The one unexpected finding was minimal discussion on the passage of ordinances banning flavored tobacco in California jurisdictions (Initiative, 2019). It could be speculated that legislators wanted to highlight the State’s role in such matters, or, possibly, avoid discussions on preemption that could detract from the larger issue. SB-793 states that greater restrictions in local ordinances cannot be superseded. Proponents of the legislation may have avoided the topic because of the exempted products in SB-793 (e.g., hookah), but, could have strengthened their case by highlighting the bill’s exclusion of the retailer exemptions found in many local ordinances (e.g., adult-only tobacco retailers).

Limitations.

While our study benefited from the timing of bills that preceded (SB-38) and occurred during the unprecedented events of 2020 (SB-793), we are limited in concluding that the reframing of the policy led to passage of the statewide tobacco flavor ban. Other factors such as the influence of key legislators (e.g., Anthony Rendon (Aguilera, 2020) may have played a greater role in the success of SB-793. Further, fewer hearings on SB-38 compared to SB-793 could account for fewer arguments regarding the impact of flavored tobacco on multiple demographic groups.

## 5 Conclusions

The reframing of flavored tobacco as a problem that impacts most age groups, not just youth, should be considered by legislators as well as community advocates. A recent example of the latter was the successful implementation of a restriction on menthol tobacco in three Minnesota cities (Bosma et al., 2021). (Bosma et al., 2021) reported that community awareness, community leadership, and conversations on racial inequities, among others, were central to the success of the policies. To counter the argument of criminalizing young Black males, the advocates in Minnesota framed the issue as a restriction on retailers, not tobacco users. Whether a tobacco flavor ban is being proposed in a city or state, it is important to emphasize the harmful impact, not culpability, on the demographic groups that use flavored tobacco.

## Funding

This research did not receive any funding from the public, commercial, or not-for-profit sector.

### CRediT authorship contribution statement

**Jasmine Tang Ker:** Conceptualization, Data curation, Formal analysis, Software, Writing – review & editing. **Natalie Delgadillo:** Data curation, Software, Validation. **Dania Amiri:** Data curation, Software, Validation. **David S. Timberlake:** Conceptualization, Methodology, Writing – original draft.

## Declaration of Competing Interest

The authors declare that they have no known competing financial interests or personal relationships that could have appeared to influence the work reported in this paper.
